# Cyclodextrins improve grape must fermentability through their detoxifying effect on medium-chain fatty acids

**DOI:** 10.1016/j.fochx.2026.103589

**Published:** 2026-02-09

**Authors:** Nadia Gregori Galán, Antoni Canalda Sabaté, Arnau Just Borràs, David García Yoldi, Nicolas Rozès, Francesca Fort, Joan Miquel Canals, Fernando Zamora, Jordi Gombau

**Affiliations:** a Universitat Rovira i Virgili, Grup d'Enologia Aplicada, Departament de Bioquímica i Biotecnologia, Facultat d'Enologia, C/ Marcel·lí Domingo S/n, 43007, Tarragona, Catalonia, Spain; b INBIOLEV, S.L., Aizoain (Navarra), Spain

**Keywords:** Medium chain fatty acids, Cyclodextrins, Alcoholic fermentation, Yeasts, Stuck fermentation

## Abstract

Through their inhibitory effects on yeast metabolism, medium-chain fatty acids (MCFAs) are one of the most frequent causes of stuck and sluggish fermentations (especially white grape must fermentation) in winemaking. A common strategy in winemaking to mitigate or correct the inhibitory effects of medium-chain fatty acids (MCFAs) on alcoholic fermentation is the addition of yeast hulls. In this paper we study α, β and γ-cyclodextrins as alternatives for mitigating the inhibitory effects of MCFAs through their ability to encapsulate non-polar molecules. Our results suggest that α-cyclodextrins encapsulate MCFAs by mitigating the toxic effects on yeast in rich MCFA media. Using α-cyclodextrins may thus be a promising strategy for mitigating the toxic effects of MCFAs and preventing sluggish and stuck fermentations in winemaking due to the presence of these compounds in the fermentation medium.

## Highlights

• Medium-chain fatty acids (MCFAs) caused sluggish and stuck alcoholic fermentation.
• MCFAs delayed yeast growth during alcoholic fermentation.
• Supplementation with **α-cyclodextrin** in an MCFA-enriched medium may neutralize the toxic effects of MCFAs.
• **α-cyclodextrin** appears to be a promising strategy for mitigating the toxicity of MCFAs and preventing sluggish and stuck fermentations in enology.

## 1 Introduction

The spontaneous stoppage of alcoholic fermentation before the fermentable sugars are fully consumed remains one of the main problems in winemaking (Alexandre & Charpentier, 1998; Bisson, 1999; Nelson & Boulton, 2024; Sablayrolles & Blateyron, 2001). This stoppage creates a high risk of developing undesirable yeasts and lactic bacteria, which can metabolize residual hexoses and significantly increase the volatile acidity of the wine (Gänzle, 2015; Van Vuuren & Dicks, 1993). The economic impact of stuck fermentation can also cause significant financial loss for producers, thus increasing winemaking costs, and reducing the quality of the final product (Zamora, 2004).

Medium-chain fatty acids (MCFA), such as octanoic (C8), decanoic (C10) and dodecanoic (C12) acids, are frequently reported to cause stuck and sluggish fermentations (Alexandre & Charpentier, 1998; Antoce et al., 2023; Banita & Antoce, 2021; Baroň, 2014; Baroň et al., 2017; Borrull et al., 2015; Lafon-Lafourcade et al., 1984; Viegas et al., 1989), especially in white grape fermentation (due to lack of oxygen, must nutritional deficiencies, and low fermentation temperatures).

The production and release of these fatty acids by yeasts during alcoholic fermentation are greater when conditions of anaerobiosis prevail and when the fermentation medium is poor in exogenous lipids (Bardi et al., 1999; Edwards et al., 1990; Restrepo et al., 2019; Valero et al., 2001).

Yeasts need to incorporate unsaturated fatty acids (UFA) into their membrane phospholipids to maintain fluidity during low-temperature fermentation (Valero et al., 2001; Weber & Bont, 1996) in a process known as homeoviscous adaptation (Los & Leusenko, 2025; Sinensky, 1974). In typical white wine fermentation, however, grape juice settling causes a deficiency in fatty acids (Bertrand & Miele, 1984), while the low-oxygen conditions prevent yeast from synthesizing UFAs and sterols (Rosenfeld & Beauvoit, 2003). As a result, the yeasts produce medium-chain fatty acids (MCFAs) to partially compensate by regulating membrane fluidity (Alexandre et al., 1996; Rozès, 1992). Some of these MCFAs are released into the medium, which can reduce yeast viability and even cause the fermentation to stop (Geneix et al., 1983).

One of the most common strategies used in winemaking to mitigate or correct the inhibitory effects of medium-chain fatty acids on alcoholic fermentation is the addition of yeast hulls. These act as adsorbents for toxic fermentation by-products, including MCFAs, and therefore reduce their inhibitory effects on fermentative yeasts (Lafon-Lafourcade et al., 1984; López et al., 2000; Muñoz & Ingledew, 1989; Muñoz & Ingledew, 1990). The adsorption mechanism is thought to be mediated by hydrophobic interactions and binding sites on the yeast cell wall that sequester these fatty acids from the fermentation medium (Pozo-Bayón et al., 2009). Besides detoxification, yeast hulls can also provide essential micronutrients such as sterols, fatty acids and nitrogenous compounds, which support yeast viability and vitality under stressful conditions (Bely et al., 2003). This dual functionality—adsorption of toxic compounds and nutrient supplementation—explains why the use of yeast hulls has become a standard enological practice for preventing or remediating the fermentation problems associated with MCFAs.

Cyclodextrins are cyclic oligosaccharides made up of glucose units. They are produced from the breakdown of starch by the enzymatic reaction of glucosyltransferase. The result is a ring-shaped molecule with a hydrophilic outer surface and a hydrophobic inner cavity. Owing to this cavity, cyclodextrin is able to form complexes with non-polar organic molecules (Astray et al., 2009; Shieh & Hedges, 1996). The most common cyclodextrins are alpha-cyclodextrin, beta-cyclodextrin and gamma-cyclodextrin, which are differentiated by having 6, 7 and 8 units of glucopyranose, respectively (Del Valle, 2004). Cyclodextrins can accommodate a wide range of chemical substances in their cavity through hydrophobic interactions, van der Waals forces or hydrogen bonds (Irwin et al., 1994; Li et al., 2024).

Their application in enology is not yet well defined, though some studies have shown that they can eliminate certain negative odors (Botelho et al., 2011; Dang et al., 2020). In this oenological context, in a recent study reported by Liu, Wei, et al., 2025 it has been described that α-CD enhanced color stability of red wine by promoting copigmentation between phenolics and monomeric anthocyanins, whereas hidroxipropil-β-CD enhanced color through direct copigmentation with anthocyanins. Moreover, Liu, Wu, et al., 2025 reported that malvidin-3O-glucoside successfully formed inclusion complexes with α-CD, improving its thermostability. In addition, Liu, Wei, et al., 2025 demonstrated that α-CD and hidroxipropil-β-CD effectively protected esters and selectively protected alcohols, improving the sensory characteristics of wine. However, their ability to encapsulate substances may help to improve the fermentability of grape musts by eliminating substances that are toxic to yeasts, such as medium-chain fatty acids. It has been reported, for example, that cyclodextrins may encapsulate fatty acids (Ondo, 2020; Pereira et al., 2022; Szejtli & Bánky-Elöd, 1975; Szente et al., 1993). In this sense, Pereira et al., 2022 demonstrated the interaction between octanoic acid and α-cyclodextrin and consequently the formation of octanoic acid/α-cyclodextrins inclusion compounds, which was studied by ^1^H NMR and isothermal titration calorimetry.

In this paper we study whether supplementation with cyclodextrins can improve the fermentability of grape must containing MCFAs. Therefore, the main goal of this study is to provide a technological approach to the effect of cyclodextrins on alcoholic fermentation behaviour based on the potential ability of cyclodextrins to encapsulate medium chain fatty acids, as previously reported in the literature (Pereira et al., 2022), thereby improving grape must fermentability.

## 2 Material and methods

### 2.1 Reagents and materials

Sodium metabisulphite (purity ≥99.5%) was purchased from Panreac (Barcelona, Spain). Octanoic acid (purity ≥99.0%), decanoic acid (purity ≥98.0%) and dodecanoic acid (purity ≥98.0%) were purchased from Merck (Barcelona, Spain). α-Cyclodextrin, β-cyclodextrin and γ-cyclodextrin were provided by Inbiolev S.L. (Aizoáin, Navarra, Spain). Rectified concentrated grape must was provided by Concentrats Pallejà (Riudoms, Tarragona, Spain).

### 2.2 Experimental design and alcoholic fermentation conditions

The fermentation medium used in this study was grape must prepared by diluting rectified concentrated must (RCM) with distilled water to achieve a potential ethanol concentration of 15.00% (*v*/v). The pH achieved for the fermentation media was 3.65. Seven liters of this grape must medium were prepared and supplemented with 50 mg/L of sulphur dioxide, added in the form of sodium metabisulfite, for use with the experimental groups.

The experimental design comprised eight experimental groups, each of which was performed in triplicate. Four experimental groups were based on grape must without MCFA supplementation: the control (C), and three groups supplemented with 200 mg/L of α-cyclodextrin (CDα), β-cyclodextrin (CDβ) or γ-cyclodextrin (CDγ), respectively. The remaining four groups were supplemented with a mixture of medium-chain fatty acids to simulate an MCFA-enriched medium. A stock ethanol-based solution containing the fatty acid mixture was prepared, and an aliquot of 0.5 mL was added to each sample to achieve final concentrations of 15 mg/L octanoic acid, 5 mg/L decanoic acid, and 1 mg/L dodecanoic acid. These concentrations are higher than those typically found in white wines that experience fermentation problems (Licek et al., 2022). One of these groups was supplemented only with the fatty acid mixture. This was designated as the negative control (FA) and used to assess the toxic effects of MCFAs on alcoholic fermentation. The other three groups (FA-CDα, FA-CDβ and FA-CDγ) were supplemented with both the fatty acid mixture and one of the different tested cyclodextrins at 200 mg/L. The aim of these latter experimental groups was to study the effect of the cyclodextrins on alcoholic fermentation in MCFA-rich media.

All fermentations were carried out in 250 mL glass flasks under controlled temperatures (18 ± 1 °C). The MCFAs and cyclodextrins were added at the beginning of fermentation prior to yeast inoculation. All experimental groups were inoculated with 200 mg/L of *Saccharomyces cerevisiae* (Uvaferm Exence™, Lallemand Inc., Montreal, Canada) to achieve an initial cell population of 6 × 10^6^ ± 1,5 × 10^6^ cell /ml in all experimental groups. This active dry yeast was hydrated according to the manufacturer's specifications.

### 2.3 Monitoring of alcoholic fermentation: Kinetics and cell yeast viability

The kinetics of alcoholic fermentation were monitored by measuring the density of the must/wine throughout the fermentation process. Density was determined using an electronic densimeter (Mettler Toledo-PortableLabTM, Cornellà de Llobregat, Barcelona, Spain).

Yeast cell counts were taken using a Neubauer chamber (Leica Microsystems GMS QmbH, Leica, Germany) and cell viability was evaluated by rhodamine B staining followed by microscopic (B–510 B F, Optika, Ponteranica, Italy) examination (Kwolek-Mirek & Zadrag-Tecza, 2014).

Aliquots from the various experimental groups were collected periodically during the alcoholic fermentation process. Depending on the cell population, samples were diluted between 10- and 100-fold with distilled water prior to analysis.

### 2.4 Chemical characterization of wines

Once fermentation was complete, the wines were supplemented with 50 mg/L of sulphur dioxide and centrifuged to remove the lees. The clarified wines were then used for chemical composition analysis. Specifically, acetic acid, glycerol, and residual sugars (glucose and fructose) were quantified using enzymatic kits and an automatic analyzer (Y15, Biosystems, Barcelona, Spain). The enzymatic kits used were acetic acid liquid (catalog number 12930), glycerol (catalog number 12812), and d-Glucose/d-Fructose (catalog number 12800), all of which were provided by Biosystems (Biosystems, Barcelona, Spain). Ethanol content was determined according to the analytical method recommended by the International Organisation of Vine and Wine (OIV, 2020).

### 2.5 Statistical analysis

All assays were performed in triplicate and the results are expressed as mean ± standard deviation of three replicates. The data obtained were subjected to statistical analysis using ANOVA and Tukey's HSD test (honestly significant difference) with XLSTAT 2020.2.3 software (Addinsoft, Paris, France). A *p*-value of less than 0.05 was considered statistically significant.

## 3 Results and discussion

### 3.1 Effect of different cyclodextrins on the kinetics and performance of alcoholic fermentation

#### 3.1.1 Effect of different cyclodextrins on the density curves during alcoholic fermentation

**Fig. 1:**
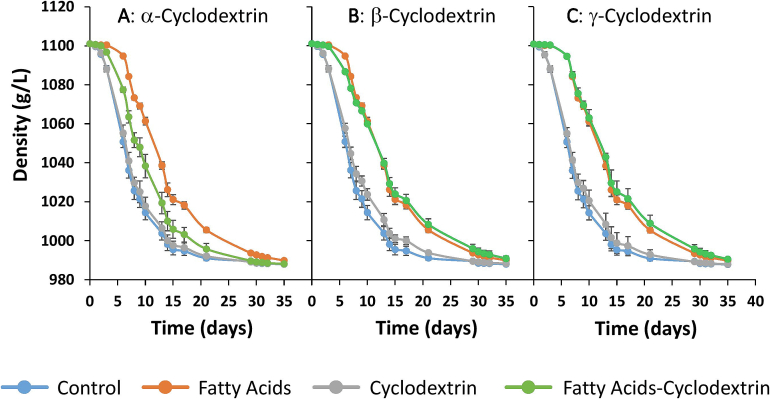
Density values during alcoholic fermentation. Control: control sample; Fatty acids: sample supplemented only with fatty acids; Cyclodextrin and fatty acid–cyclodextrin: correspond to samples containing the different cyclodextrins, without or with fatty acids, respectively. All the figures (1A, 1B and 1C) show the control and fatty acid samples. Figures 1A, 1B and 1C show the samples containing α-cyclodextrin, β-Cyclodextrin and γ-Cyclodextrin, respectively.

Fig. 1 shows the evolution of must/wine density throughout the alcoholic fermentation process to evaluate the effect of different cyclodextrins on fermentation kinetics. Specifically, Figs. 1.A, 1.B, and 1.C illustrate the effects of α-cyclodextrin, β-cyclodextrin, and γ-cyclodextrin, respectively, on the density curves. These density curves allow indirect assessment of the fermentation rate since a decrease in this physical parameter is associated with sugar consumption by yeast during alcoholic fermentation.

As Figs. 1.A, 1.B and 1.C show, the control sample recorded the highest fermentation rate and the FA group recorded the slowest. This suggests that a delay in fermentation rate was caused by the presence of MCFAs in the medium.

The control sample reached its lowest density on day 30 and this value remained stable until day 35. The density value of the FA sample on day 35 was slightly but statistically higher than that of the control. This suggests a stuck fermentation in the FA sample, which, as is reported in the literature (Antoce et al., 2023; Borrull et al., 2015; Viegas et al., 1989), can be attributed to the toxic effect of MCFAs on yeast metabolism.

The samples supplemented with the cyclodextrins (α-CD, β-CD, γ-CD) in a non-enriched MCFA medium exhibited fermentation rates similar to that of the control sample. However, a slight delay in the fermentation rate was observed for the β-CD and γ-CD samples, though this difference was not significant. This delay may be due to the encapsulation of essential nutrients for β-cyclodextrin and γ-cyclodextrin, which impairs yeast metabolism. However, these samples achieved similar density values to that of the control on day 35 of alcoholic fermentation, which indicates that alcoholic fermentation was successfully completed for these samples.

A different trend was observed in the samples containing the various cyclodextrins in an MCFA-enriched medium (FA-α-CD, FA-β-CD, FA-γ-CD). The presence of β-cyclodextrin and γ-cyclodextrin combined with MCFA supplementation caused a delay in the fermentation rate similar to that observed in the FA sample. Moreover, the density values for these samples were similar to those of the FA sample on day 35, which further suggests the occurrence of stuck alcoholic fermentation. On the other hand, the presence of α-cyclodextrin in the enriched MCFA medium (Fig. 1.A) caused a delay in the fermentation rate in the early stages of alcoholic fermentation but this later accelerated, reaching density values on day 30 that were similar to those of the control and the α-CD, β-CD, and γ-CD samples. This suggests that MCFAs in the medium cause delays in the fermentation rate due to the toxic effects attributed to these compounds in the literature. However, it appears that α-cyclodextrin is able to encapsulate these MCFAs, as it is reported in the literature (Pereira et al., 2022), thus improving fermentation performance and kinetics. In contrast, β-cyclodextrin and γ-cyclodextrin do not seem to mitigate the toxic effect of MCFAs. The capacity of cyclodextrins to encapsulate MCFAs thus appears to be influenced by their chemical structure, specifically, by the number of glucose units of which they are composed.

#### 3.1.2 Effect of different cyclodextrins on the area under the density curves and the glucose and fructose concentration of final wines

To quantitatively assess the kinetics of alcoholic fermentation, we calculated the area under the density curves since this reflects the rate of sugar consumption over time and, therefore, the fermentation rate. In this context, a smaller area indicates a faster fermentation rate and therefore better fermentation performance. We also measured glucose and fructose concentrations to determine whether a stuck fermentation had occurred. Sugar levels were analyzed on day 35 of alcoholic fermentation since the density values had remained constant for three consecutive days.

Table 1 summarizes the calculated areas and the concentrations of glucose and fructose for all experimental groups.

**Table 1:** Area under the density curve and concentration of glucose and fructose of the wines at end of alcoholic fermentation.

**Sample**	AUDC				Glucose/Fructose (g/L)			
**C**	116	±	4	**A**	0.89	±	0.21	**A**
**α-CD**	118	±	2	**A**	0.75	±	0.13	**A**
**β-CD**	124	±	3	**A**	0.98	±	0.23	**A**
**γ-CD**	124	±	5	**A**	0.96	±	2.79	**A**
**FA**	147	±	3	**C**	4.89	±	0.89	**B**
**FA-α-CD**	134	±	3	**B**	0.81	±	0.10	**A**
**FA-β-CD**	154	±	3	**C**	7.16	±	3.19	**B**
**FA-γ-CD**	150	±	11	**C**	7.23	±	2.39	**B**

AUDC: Area under the density curve. C: control, α- CD, β-CD and γ-CD: samples supplemented with α, β and γ- cyclodextrins respectively. FA: sample supplemented with medium chain fatty acids. FA-α- CD, FA-β-CD and FA-γ-CD: samples supplemented with medium chain fatty acids and the different cyclodextrins. Different letters indicate statistically differences between the different conditions at *p* value <0.05.

On one hand, the control sample recorded the lowest area under the density curve, though this was statistically similar to those of the α-CD, β-CD, and γ-CD samples. These results confirm that the fermentation kinetics of these samples were similar to those of the control. The slight delay relative to the control observed in Figs. 1.B and 1.C for the β-CD and γ-CD samples, respectively, was not statistically significant since no statistically significant differences were observed in terms of the areas under the density curves. The presence of the various cyclodextrins in a non-enriched MCFA medium therefore had no significant effect on alcoholic fermentation kinetics.

The glucose and fructose concentrations for all these samples were below 1 g/L, which indicates that fermentation was successfully completed. These findings confirm that the cyclodextrins in non-enriched MCFA media had no statistically significant impact on alcoholic fermentation performance compared to the control.

On the other hand, the FA sample exhibited a statistically higher area under the density curve in comparison with the control sample. This increase is attributed to a lower fermentation rate and a possible stuck or sluggish fermentation (Fig. 1). The glucose and fructose concentrations (4.89 g/L) measured on day 35 for the FA sample thus confirmed the hypothesized stuck fermentation seen in Fig. 1. These findings support the previously described toxic effect of medium-chain fatty acids on yeast metabolism. In this context, the presence of MCFAs in the medium led to a sluggish and ultimately stuck alcoholic fermentation.

The presence of cyclodextrins in the enriched MCFA media led to differences in the kinetics and performance of alcoholic fermentation. Specifically, for samples FA-β-CD and FA-γ-CD, the area under the density curve and the glucose and fructose concentrations were statistically similar to those of the FA sample (Table 1). These findings corroborate the results observed in Fig. 1 and thus confirm that the presence of β-cyclodextrin and γ-cyclodextrin in the enriched MCFA media did not improve the alcoholic fermentation kinetics. The presence of these cyclodextrins did not prevent the appearance of also stuck fermentation since the results were similar to those observed for the FA sample.

In contrast, the FA-α-CD sample recorded a lower area under the density curve than the FA sample, which suggests that the presence of α-cyclodextrin improved the kinetics of alcoholic fermentation. However, this area was still slightly higher than that of the control sample, which indicates that fermentation in the FA-α-CD sample proceeded more slowly than in the control. However, on day 35 of alcoholic fermentation, the density values of the FA-α-CD sample were similar to those of the samples without MCFA, which indicates that no stuck fermentation occurred in this sample. These results suggest that the presence of MCFA caused a delay in the early stages of fermentation. However, the presence of α-cyclodextrin appears to accelerate the fermentation process in an MCFA-enriched medium, probably due to its ability to encapsulate fatty acids, as it is reported in the literature (Pereira et al., 2022). This encapsulation may have prevented fermentation inhibition by mitigating the toxic effects of MCFAs, thereby preventing a stuck fermentation in the FA-α-CD sample.

These findings suggest that α-cyclodextrin is able to encapsulate MCFAs in the medium, thus mitigating their toxic effects on *Saccharomyces cerevisiae* during fermentation. Conversely, β- and γ-cyclodextrins seem less effective in encapsulating these fatty acids. The chemical structure of cyclodextrins, particularly their number of glucose units, may therefore play a critical role in determining their capacity to bind and neutralize the toxic effects of MCFAs.

#### 3.1.3 Effect of different cyclodextrins on the yeast population during alcoholic fermentation

**Fig. 2:**
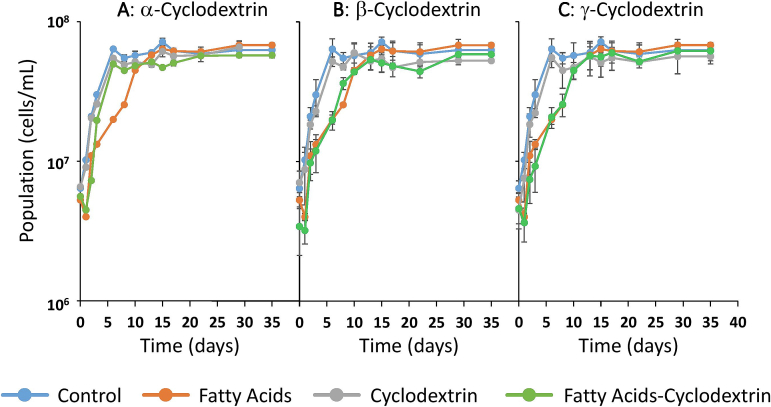
Total yeast population during alcoholic fermentation. Control: control sample; Fatty acids: sample supplemented only with fatty acids; Cyclodextrin and fatty acid–cyclodextrin: correspond to samples containing the different cyclodextrins, without or with fatty acids, respectively. All the figures (2A, 2B and 2C) show the control and fatty acid samples. Figures 2A, 2B and 2C show the samples containing α-cyclodextrin, β-Cyclodextrin and γ-Cyclodextrin, respectively.

**Fig. 3:**
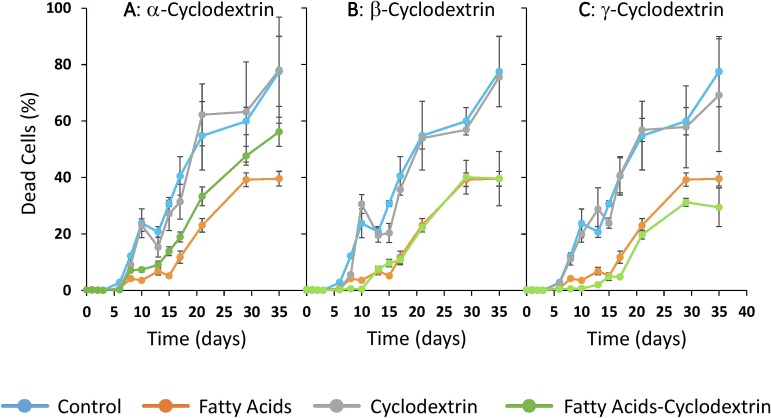
Percentage of yeast mortality. Control: control sample; Fatty acids: sample supplemented only with fatty acids; Cyclodextrin and fatty acid–cyclodextrin: correspond to samples containing the different cyclodextrins, without or with fatty acids, respectively. All the figures (3A, 3B and 3C) show the control and fatty acid samples. Figures 3A, 3B and 3C show the samples containing α-cyclodextrin, β-Cyclodextrin and γ-Cyclodextrin, respectively.

Fig. 2 shows the total yeast cell count (viable and dead cells) throughout the alcoholic fermentation, while Fig. 3 shows the percentage of dead cells relative to the total cell population.

In the case of the control sample, the total cell population increased exponentially from the first day of alcoholic fermentation until day 5. This increase corresponds to viable cells, as no dead cells were detected during the first five days of fermentation (Fig. 3). This increase in viable cells is consistent with the expected exponential growth of yeast typically observed in grape must media.

From day 5 of alcoholic fermentation, the total cell population in the control sample remained stable until the end of alcoholic fermentation (day 35). However, as Fig. 3 shows, the percentage of dead cells increased significantly from day 5 until the end of alcoholic fermentation. This suggests a progressive decline in cell viability from day 5 until the end of alcoholic fermentation. This expected decline in viability is probably due to cell death caused by stress factors described in the literature (Bisson, 1999), such as high ethanol concentrations.

At the onset of alcoholic fermentation, the FA sample exhibited a delayed growth in the total yeast population as well as a longer lag phase compared to the control. As in the control sample, this initial growth was attributed to viable cells, since no dead cells were observed at the beginning of fermentation (Fig. 3). This delayed growth was probably caused by the presence of MCFAs in the medium, which are known to exert an inhibitory effect on yeast metabolism and growth (Viegas et al., 1989).

The exponential growth phase of yeast in the FA sample lasted approximately 15 days, which is significantly longer than in the control. From day 15 onward, the total cell population remained stable while the proportion of dead cells increased progressively.

The presence of cyclodextrins in the non-MCFA-enriched media (samples α-CD, β-CD, γ-CD) resulted in similar yeast growth trends to that of the control sample. This observation is consistent with the density data shown in Fig. 1, which indicate similar fermentation performance under these conditions. However, several differences were observed in the samples containing cyclodextrins in the MCFA-enriched media. Specifically, the sample containing MCFAs and α-cyclodextrin exhibited a delayed yeast growth at the onset of alcoholic fermentation that was comparable to that observed in the FA sample. However, from day 3 onwards, yeast growth in this sample accelerated significantly in comparison with the FA sample, achieving a viable cell population by day 5 that was slightly lower than the control but markedly higher than that of the FA sample. These results suggest that MCFAs exert an inhibitory effect on yeast growth during the initial stages of alcoholic fermentation. However, the presence of α-cyclodextrin appears to mitigate this toxic effect, probably by encapsulating the MCFAs, as it is described by Pereira et al., 2022, and thus promoting yeast growth under these otherwise inhibitory conditions.

The presence of β- and γ-cyclodextrins in the MCFA-enriched media (samples FA-β-CD and FA-γ-CD) exerted a similar trend to that of the FA in terms of total cell population and the percentage of dead cells. This suggests that β- and γ-cyclodextrins did not mitigate the toxic effects of MCFAs on yeast growth. These findings are also consistent with the trends in the fermentation rate shown in Fig. 1.

Surprisingly, the percentage of dead yeast cells increased more rapidly during alcoholic fermentation in the samples that were not enriched with MCFAs. Moreover, on day 35 the proportion of dead cells was higher in samples without MCFAs than in those containing MCFAs. This unexpected observation may be attributed to the fact that the samples without MCFA supplementation reached higher ethanol concentrations earlier, thus potentially inducing stress conditions that accelerated cell death.

At the end of alcoholic fermentation, all samples without MCFAs showed statistically similar percentages of dead yeast cells. In contrast, the FA, FA–β-CD, and FA–γ-CD samples also showed similar percentages of dead cells. However, these values were significantly lower than those observed in the samples without MCFAs, as previously described.

However, the percentage of dead cells in the FA–α-CD sample at the end of alcoholic fermentation was statistically higher compared to the other MCFA-containing samples and statistically similar (though slightly lower) compared to the non-MCFA-containing samples. These results suggest that the presence of MCFAs in the medium did not lead to increased yeast cell death and that MCFAs exert an inhibitory effect on yeast metabolism without compromising cell viability.

### 3.2 Effect of cyclodextrins on the chemical composition of wines; ethanol, acetic acid and glycerol concentrations

Table 2 shows the ethanol content and concentrations of glycerol and acetic acid in the final wines after 35 days of alcoholic fermentation. No significant differences between the experimental groups were observed in ethanol content. Although stuck fermentation was detected in the FA, FA–β-CD and FA–γ-CD samples, as evidenced by the presence of residual sugars in these samples (Table 1), no statistically significant decrease in ethanol content was found in these wines in comparison with the other experimental groups (control, a-CD β-CD, γ-CD).

**Table 2:** Ethanol content, glycerol and acetic acid concentrations of different final wines.

**Sample**	Ethanol content (% v/v)				Glycerol (g/L)				Acetic acid (g/L)			
**C**	16.1	±	0.1	**A**	6.97	±	0.25	**B**	0.46	±	0.02	**A**
**α-CD**	16.1	±	0.2	**A**	7.23	±	0.58	**B**	0.50	±	0.02	**AB**
**β-CD**	16.0	±	0.3	**A**	7.17	±	0.29	**B**	0.56	±	0.05	**AB**
**γ-CD**	16.3	±	0.1	**A**	6.87	±	0.38	**B**	0.52	±	0.02	**A**
**FA**	15.8	±	0.2	**A**	5.77	±	0.09	**A**	0.71	±	0.01	**C**
**FA-α-CD**	16.2	±	0.2	**A**	6.07	±	0.25	**A**	0.60	±	0.09	**B**
**FA-β-CD**	16.0	±	0.2	**A**	6.00	±	0.08	**A**	0.74	±	0.03	**C**
**FA-γ-CD**	16.0	±	0.3	**A**	6.00	±	0.24	**A**	0.76	±	0.03	**C**

C: control, α- CD, β-CD and γ-CD: samples supplemented with α, β and γ- cyclodextrins respectively. FA: sample supplemented with medium chain fatty acids. FA-α- CD, FA-β-CD and FA-γ-CD: samples supplemented with medium chain fatty acids and the different cyclodextrins. Different letters indicate statistically differences between the different conditions at *p* value <0.05.

Note that no statistically significant differences were observed in ethanol content between the samples that experienced a stuck fermentation (FA, FA–β-CD and FA–γ-CD) and the other samples, despite the latter having metabolized a higher sugar concentration. One explanation for this is that the yeasts in the samples that completed fermentation may have directed some of the sugars through the glyceropyruvic metabolic pathway (Zamora, 2009). All samples containing MCFAs thus exhibited significantly lower glycerol levels than those fermented in a medium that was not enriched with MCFAs. This may explain the absence of significant differences in ethanol content among these samples despite a lower amount of metabolized sugars.

Finally, except for the FA–α-CD sample, the highest concentrations of acetic acid were observed in the samples containing MCFAs. These results suggest that the presence of MCFAs in the fermentation medium induces stress conditions on yeast, thereby leading to increased acetic acid production. However, the comparatively lower concentration of acetic acid in the FA–α-CD sample suggests that α-cyclodextrin mitigates this stress. This is probably due to their ability to encapsulate MCFAs present in the medium, as it is reported in the literature (Pereira et al., 2022), thus reducing their toxic effects and alleviating the associated stress conditions during alcoholic fermentation. However, the concentration of acetic acid was slightly (but statistically) higher in the FA–α-CD sample than in the samples that did not contain MCFAs, whose concentrations were all statistically similar. Therefore, since acetic acid is regarded as an off-odor compound in enology, using α-cyclodextrins may be a promising strategy for reducing acetic acid production by yeast in MCFA-enriched media.

## 4 Conclusion

Our results confirm that MCFAs present in the medium induce stress on *Saccharomyces cerevisiae* metabolism due to their toxic effects previously described in the literature. MCFAs lead to sluggish and stuck fermentations, resulting in residual sugars in the wine and higher levels of acetic acid. MCFAs do not appear to cause yeast cell death but delay their growth and reduce yeast proliferation during the fermentation process. However, the use of cyclodextrins—specifically α-cyclodextrins—in an MCFA-rich medium may neutralize the toxic effects of MCFAs, leading to faster and more successful fermentations. This suggests that α-cyclodextrin is able to encapsulate MCFAs and thus mitigate their toxicity. Importantly, β- and γ-cyclodextrins did not alleviate this toxic effect, which indicates that the chemical structure of cyclodextrins largely determines their capacity to encapsulate MCFA molecules.

The use of α-cyclodextrins appears to be a promising strategy for mitigating the toxic effects of MCFAs and preventing sluggish and stuck fermentations in enology caused by these compounds in the fermentation medium. Since the effect of cyclodextrins was not observed in media without MCFAs, further studies are needed under more realistic enological conditions, where *Saccharomyces cerevisiae* produces MCFAs endogenously and the MCFAs concentrations are more representative of those found in wine. In line with this perspective, further studies are also needed to evaluate the effect of α-cyclodextrins on the sensory characteristics of wine, given their potential interactions with volatile compounds present in wine, as previously reported in the literature by Liu et al., 2025.

## CRediT authorship contribution statement

**Nadia Gregori Galán:** Writing – original draft, Methodology, Investigation. **Antoni Canalda Sabaté:** Methodology, Investigation. **Arnau Just Borràs:** Methodology, Investigation. **David García Yoldi:** Visualization, Supervision. **Nicolas Rozès:** Visualization, Supervision. **Francesca Fort:** Visualization, Supervision. **Joan Miquel Canals:** Visualization, Supervision. **Fernando Zamora:** Writing – review & editing, Writing – original draft, Visualization, Supervision, Methodology, Investigation, Conceptualization. **Jordi Gombau:** Writing – review & editing, Writing – original draft, Visualization, Supervision, Methodology, Investigation, Conceptualization.

## Declaration of competing interest

The authors declare that they have no known competing financial interests or personal relationships that could have appeared to influence the work reported in this paper.

## Data Availability

Data will be made available on request.
